# Ubiquitin Carboxyl-Terminal Hydrolase L1 of Cardiomyocytes Promotes Macroautophagy and Proteostasis and Protects Against Post-myocardial Infarction Cardiac Remodeling and Heart Failure

**DOI:** 10.3389/fcvm.2022.866901

**Published:** 2022-04-07

**Authors:** Penglong Wu, Yifan Li, Mingqi Cai, Bo Ye, Bingchuan Geng, Faqian Li, Hua Zhu, Jinbao Liu, Xuejun Wang

**Affiliations:** ^1^Division of Basic Biomedical Sciences, University of South Dakota Sanford School of Medicine, Vermillion, SD, United States; ^2^Department of Cardiology, Xiamen Cardiovascular Hospital of Xiamen University, School of Medicine, Xiamen University, Xiamen, China; ^3^Lillehei Heart Institute and the Department of Medicine, University of Minnesota College of Medicine, Minneapolis, MN, United States; ^4^Department of Surgery, The Ohio State University Wexner Medical Center, Columbus, OH, United States; ^5^Department of Laboratory Medicine and Pathology, University of Minnesota Medical School, Minneapolis, MN, United States; ^6^Guangzhou Municipal and Guangdong Provincial Key Laboratory of Protein Modification and Degradation, School of Basic Medical Sciences, Guangzhou Medical University, Guangzhou, China

**Keywords:** autophagy, cardiac remodeling, cardiomyocyte, myocardial infraction, Uchl1, ubiquitin, mice, humans

## Abstract

Ubiquitin carboxyl-terminal hydrolase L1 (UCHL1) is a deubiquitinase known to play essential roles in the nervous tissue. Myocardial upregulation of UCHL1 was observed in human dilated cardiomyopathy and several animal models of heart disease, but the (patho)physiological significance of UCHL1 in cardiomyocytes remains undefined. Hence, we conducted this study to fill this critical gap. We produced cardiomyocyte-restricted *Uchl1* knockout (CKO) by coupling the *Uchl1*-floxed allele with transgenic *Myh6-Cre* in C57B/6J inbred mice. Mice transgenic for *Myh6-Cre* were used as controls (CTL). Myocardial Uchl1 proteins were markedly reduced in CKO mice but they did not display discernible abnormal phenotype. Ten-week old CTL or CKO mice were subjected to left anterior descending artery ligation (myocardial infarction, MI) or sham surgery (Sham) and characterized at 7- and 28-day after surgery. Compared with Sham mice, significant increases in myocardial UCHL1 proteins were detected in CTL MI but not in CKO MI mice. MI-induced left ventricular (LV) chamber dilation, reduction of ejection fraction (EF) and fractional shortening (FS), and LV anterior wall thinning detected by echocardiography were comparable between the CTL MI and CKO MI groups 7-day post-MI. However, by 28-day post-MI, MI-induced LV chamber dilatation, EF and FS reduction, increases of myocardial ubiquitin conjugates, and increases in the heart weight to body weight ratio and the ventricular weight to body weight ratio were significantly more pronounced in CKO MI than CTL MI mice. As further revealed by LV pressure-volume relationship analyses, CKO MI mice but not CTL MI mice displayed significant decreases in stroke volume, cardiac output, and the maximum rates of LV pressure rising or declining and of LV volume declining, as well as significant increases in LV end-diastolic pressure and Tau, compared with their respective Sham controls. LC3-II flux assays reveal that autophagic flux is decreased in CKO mouse myocardium as well as in cultured Uchl1-deficient cardiomyocytes. In conclusion, UCHL1 of cardiomyocytes is dispensable for development but promotes macroautophagy in cardiomyocytes. Upregulation of UCHL1 in post-MI hearts occurs primarily in the cardiomyocytes and protects against post-MI cardiac remodeling and malfunction likely through supporting autophagic flux and proteostasis during a stress condition.

## Introduction

Ubiquitination is arguably the most prevalent posttranslational modification in the cell and regulates virtually all cellular processes either through the ubiquitin-proteasome system (UPS)-mediated degradation of the modified proteins or by changing directly the activity and interactomes of the modified proteins (1). Ubiquitination is the process that covalently attaches a ubiquitin (Ub) or a chain of Ub to a substrate protein *via* an isopeptide bond. This process involves a cascade of biochemical reactions catalyzed sequentially by the Ub activating enzyme (E1), Ub conjugating enzymes (E2), and Ub ligases (E3) in a substrate protein-specific manner. The substrate specificity of ubiquitination is conferred by the E3 (2). Ubiquitination is reversible and the reversed process, known as deubiquitination, is catalyzed by deubiquitinases (DUBs), which cleaves the Ub or Ub chain from the substrate and dissembles Ub chains. The human genome encodes approximately 100 DUBs, but most of them have not been well studied for their biology and physiology (3). Ub carboxyl-terminal hydrolase L1 (UCHL1), also known as PGP9.5 or PARK5, is a DUB of the UCH family (4). UCHL1 is known to be highly expressed and play an essential role in neurons; hence, it has been commonly used as a neuronal marker (5, 6). Mutations in the *UCHL1* gene are linked to neurodegenerative disease, such as an early-onset progressive neurodegeneration syndrome (7) and familial Parkinson’s disease (8). UCHL1 gain-of-function has been connected to the genesis of various types of tumor, including breast cancer (9), ovarian cancer (10), and lymphoma (11); hence, UCHL1 is regarded as an oncogene (11). Accordingly, inhibition of UCHL1 is being intensively studied as a potential anticancer strategy, which is further fueled by the commercial availability of UCHL1-specific chemical inhibitors (6, 9, 12). UCHL1 inhibitors are also being tested for their anti-fibrotic effects (12–15). Therefore, it becomes highly relevant to study the impact of UCHL1 loss of function on the heart.

The pathophysiological significance of UCHL1 in the heart has been implicated by the observation that myocardial UCHL1 mRNA and protein levels are significantly upregulated in human dilated cardiomyopathy (16), as well as in animal models of several cardiac pathological conditions including myocardial infarction (MI) induced surgically by left anterior descending artery (LAD) ligation (15, 17), right ventricular pressure overload induced by pulmonary artery constriction (18), left ventricular (LV) pressure overload induced by transverse aortic constriction (TAC) (19, 20), and myocarditis induced by coxsackievirus infection (21). Bi et al. reported that global inhibition of UCHL1 using heterozygous germ-line Uchl1 knockout or a pharmacological compound (LDN-57444) attenuated TAC-induced cardiac hypertrophy, fibrosis, and LV malfunction in mice and, conversely, overexpression of UCHL1 *via* recombinant serotype 9 adeno-associated viruses (rAAV9)-mediated gene delivery exacerbated the pathology (20). This same group subsequently reported similar findings in the spontaneous hypertensive rat model where only pharmacological inhibition of UCHL1 by LDN-57444 was used (22). Lei et al. reported that administration of LDN-57444 attenuated myocardial fibrosis and LV malfunction in post-MI mice (15). More recently, another group reported that systemic administration of LDN-57444 inhibited angiotensin II infusion-induced cardiac fibrosis (14). These recent reports seem to suggest that myocardial upregulation of UCHL1 is detrimental to the heart in both pressure overload and post-MI settings.

Notably, either global Uchl1-KO or the systemic administration of a UCHL1 inhibitor was used by the cardiac studies highlighted above for UCHL1 inhibition, but both approaches suppress UCHL1 indiscreetly in all cell types and all organs. Hence, the (patho)physiological significance of UCHL1 in the cardiomyocyte compartment remains undefined. To fill this critical gap, we created mice with cardiomyocyte-restricted knockout of *Uchl1* (Uchl1-cko) for the present study to test the **hypothesis** that UCHL1 in cardiomyocytes is dispensable for cardiac development but plays an important role in maintaining proteostasis in diseased hearts. Our study provides the first genetic evidence that UCHL1 in cardiomyocytes is dispensable for perinatal cardiac development, but post-MI myocardial upregulation of UCHL1 occurs primarily in cardiomyocytes in both mice and humans and this upregulation is indispensable for cardiac proteostasis and protects against post-MI cardiac remodeling and heart failure.

## Materials and Methods

### Animals

The care and use of animals in this study followed NIH guidelines and were reviewed and approved by the University of South Dakota Institutional Animal Care and Use Committee. We have previously reported the creation of the mouse harboring a *Uchl1*-floxed allele (*Uchl1^flox/flox^*), where exon 2 of the *Uchl1* gene is floxed (23). The creation of the transgenic mice overexpressing the *Cre* recombinase under the control of the mouse α-myosin heavy chain promoter (Myh6-Cre) was previously described (24). Both *Uchl1^flox/flox^* mice and Myh6-Cre transgenic mice used in this study are in the C57BL/6J inbred background. Homozygous cardiomyocyte-restricted *Uchl1* knockout (*Uchl1*^flox/flox^*::Myh6-Cre*; hereafter referred to as Uchl1-cko or CKO) mice were generated by cross-breeding *Uchl1*^flox/flox^** with hemizygous transgenic Myh6-Cre. Mice with a genotype of *Uchl1^+/+^::Myh6-Cre* were used as the control genotype (CTL).

### Myocardial Infarction Model

Myocardial infarction was produced *via* surgical ligation of the left anterior descending coronary artery (LAD) in 10-week old mice of both sexes under aseptic conditions as previously described with minor modifications (25). CTL and CKO mice were randomly divided into two groups: one subject to LAD ligation (MI) and the other subjected to sham surgery (Sham). The surgeon was blinded to mouse genotypes. The animal was exposed to 4.5% isoflurane before intubated *via* the mouth and mechanically ventilated with a ventilator (MiniVent Type 845, Harvard Apparatus). During the surgery anesthesia was maintained with 1.5% isoflurane in medical oxygen with a tidal volume of 200–250 μL and a frequency of 130–150 strokes/min. After hair removal and skin disinfection, a thoracotomy was performed at the forth intercostal space to expose the beating heart. The LAD was ligated with an 8-0 suture to induce MI. The same surgical procedure but without the LAD ligation step was performed for the shame control group. After LAD ligation or sham surgery, the chest was closed with a 4-0 silk suture and skin was sealed, then the trachea cannula was gently removed. Buprenorphine SR (1 mg/kg) was administrated immediately after the surgery was completed and anesthesia was withdrawn and before the animal was placed on a warm surface in a new clean cage for recovery.

### Echocardiography

Trans-thoracic echocardiography (Echo) of post-surgery mice at different time points was performed using the VisualSonics Vevo 2100 system and a 40 MHz probe as previously described (26). Mice were kept in light anesthesia with inhalation of 1.5% isoflurane in room air supplemented with 100% oxygen when echocardiographs were recorded. A 2D-mode long axis view of LV was recorded and 2D-guilded M-Mode echocardiographs through the anterior and posterior walls were acquired. Primary measurements of the end-diastolic and the end-systolic LV anterior and posterior wall thickness, LV end-diastolic and end-systolic chamber dimensions, and heart rate (HR) were used to derive functional parameters, such as percentage fractional shortening (FS), ejection fraction (EF), stroke volume (SV), and cardiac output (CO) as previously reported (27). Blinding procedures were applied to mouse Echo-based experiments. The echocardiographer was blinded to mouse genotypes and experimental groups during Echo recording and subsequent Echo measurements.

### Left Ventricular Pressure–Volume Relationship Analysis

Left ventricular pressure–volume relationship (P-V loop) analyses were performed as previously described (25). At the terminal experiment, mice were anesthetized *via* inhalation of 4.5% isoflurane in medical grade oxygen and sustained *via* inhalation of 1.5% isoflurane. After orally intubated, mice were mechanically ventilated at a tidal volume of ∼200 μl and a frequency of 130–150 strokes/min. Body temperature was monitored with a rectal thermometer and maintained at 37°C. A 1.4 F Millar Mikro-Tip catheter P-V transducer (SPR-895, Millar Instruments) was gingerly inserted into the needle-prepunched right common carotid artery and advanced to the LV chamber. After stabilizing for 30 min, LV P-V loops were recorded using a Powerlab data acquisition system (AD Instruments), and all parameters were derived using the associated software. Both saline calibration and cuvette calibration were applied before data exportation. Blinding procedures were applied to LV P-V loop analysis. The person collecting the P-V loops and subsequently taking the measurements was blinded to mouse genotypes and group assignment.

### Neonatal Mouse Cardiomyocyte Culture and Adenoviral *Cre* Mediated *Uchl1* Knockout

Primary neonatal mouse ventricular myocytes (NMVMs) were isolated from the ventricles of 2-day old *Uchl1^flox/flox^* or wild type (WT) mice and plated on 6-cm Petri dishes at a density of 2.0 × 10^6^ cells in DMEM with 10% FBS as previously described (28). The plated cells were then cultured in a 5% CO2 incubator at 37°C for at least 24 h before the medium was changed to meet the needs of subsequent experiments. Forty-eight hours after plating, cells were infected with recombinant replication-deficient adenoviruses harboring the *Cre* expression cassette for 4 h in DMEM media. Post-infection cells were maintained in 2% FBS, 1% penicillin/streptomycin in DMEM until subsequent treatments.

### Western Blot Analysis

The extraction of total proteins from ventricular myocardial samples was done using 1× loading buffer containing 41 mM Tris–HCl, 1.2% sodium dodecyl sulfate (SDS), and 8% glycerol. A cocktail of protease inhibitors (#P-1540, AG Scientific, CA, United States) were added to the extraction buffer to inhibit protein degradation. Protein concentration was determined using bicinchoninic acid reagents (#23225, ThemoFisher Scientific, Waltham, MA, United States). Equal amounts of proteins were fractionated *via* 8–14% SDS polyacrylamide gel electrophoresis (SDS-PAGE) and the separated proteins were transferred onto polyvinylidene difluoride (PVDF) membrane using a trans-blot apparatus (Bio-Rad, Hercules, CA, United States). The SDS-PAGE gel contained 0.5% 2,2,2-Trichloroethanol (T54801, Sigma) which enables stain-free imaging of proteins in the gel and on the PVDF membrane for a better in-lane loading control for subsequent immunoblot analyses (29). The PVDF membranes were then sequentially subject to blocking, incubation with the primary antibodies against the protein of interest (see Supplementary Table 1), washing with TBST buffer to remove unbound primary antibodies, incubation with horseradish peroxidase (HRP) conjugated secondary antibodies (Santa Cruz Biotechnology), and washing to remove unbound antibodies. The secondary antibodies bound to the PVDF membrane were then detected using enhanced chemiluminescence reagents (GE Healthcare, NJ, United States); the chemiluminescence was digitally imaged and analyzed with the ChemiDoc™ MP imaging system and associated software (Bio-Rad, Hercules, CA, United States) as we previously reported (26).

### Autophagic Flux Assays in Mice and Cardiomyocytes

Targeted mice were intraperitoneally injected with bafilomycin-A1 (BFA1, 1.868 mg/kg body weight) (Item #11038, Cayman Chemical, Ann Arbor, MI, United States) to inhibit lysosomal degradation or with vehicle control (dimethyl sulfoxide, DMSO). Ventricular myocardium was sampled from the mice 1 h after the injection, for Western blot analyses for LC3-II levels as previously described (25). For *in vitro* assays, The cultured NMVMs were treated with BFA1 (6 nM) or DMSO for 24 h before they were harvested for extraction of total proteins with the 1× loading buffer as previously described (28). Western blot analyses for LC3-II levels were performed. The LC3-II flux presented here refers to the net amount of LC3-II accumulated by the BFA1-mediated lysosomal inhibition. Mathematically, it is calculated by subtracting the GAPDH normalized LC3-II level of a BFA-treated sample with the mean value of the GAPDH normalized LC3-II levels of the DMSO treated samples of the same genotype group.

### Immunofluorescence Staining and Confocal Microscopy

Perfusion-fixation and cryosectioning of mouse heart tissues were performed as previously described (28). The distribution of Uchl1 in myocardium was visualized with indirect immunofluorescence staining followed by confocal microscopy using a Zeiss confocal microscope (LSM780). Co-immunostaining of sarcomeric α-actin was utilized to identify cardiomyocytes. Primary antibodies used were rabbit monoclonal anti-UCHL1 (#13179, Cell Signaling Technology) and mouse monoclonal anti-sarcomeric α-actin antibody (#A2172, Sigma); secondary antibodies included anti-rabbit IgG Alexa Fluor 546 (A10040, Invitrogen) and Anti-mouse IgM Alexa Fluor 488 (A21042, Invitrogen).

The human ventricular myocardial specimen used here originated from de-identified human non-failing donor hearts (NFH) failed matching for appropriate recipients or from the tissue cylinder core obtained from de-identified patients during the installation of a LV assisting device (LVAD). For the latter, acute myocardial infarction (AMI) was verified by H&E histological examination and dated from 1 to 7 days post AMI. The research protocol (#STUDY00013028) was exempted by the Institutional Review Board of the University of Minnesota. Formalin-fixed paraffin-embedded 4-μm sections of the human myocardium were deparaffinized and rehydrated. Microwave-citrate antigen retrieval was performed at 95–99°C for 20 min using sodium citrate buffer (10 mM Sodium citrate, 0.05% Tween 20, pH 6.0). Tissue slides were then blocked with a blocking/permeabilization solution (0.3% Triton X-100 and 1% bovine serum albumin in PBS), incubated with primary antibodies against cardiac troponin T (cTnT) (MA5-12960, Thermo Fisher) or against UCHL1 (#13179, Cell Signaling Technology) overnight at 4°C, followed by incubation with Dylight-488 and 555 secondary antibodies (Invitrogen) for 45-min. Sections were counterstained with DAPI, mounted with coverslips using Fluoromount-G Mounting Medium (Thermo Fisher) and imaged using a Nikon E800 spinning disk confocal microscope.

### Statistical Methods

The GraphPad Prism software (Version 8.4; GraphPad Software, San Diego, CA, United States) was used. All continuous variables are presented as scatter dot plots with mean ± SEM superimposed. All data were examined for normality with the Shapiro–Wilk’s test prior to application of parametric statistical tests. Those that failed this test were analyzed with the Mann–Whitney test. Tests used for statistical significance evaluations of each data set are specified in figure legends. Difference between two groups was evaluated using two-tailed unpaired Student’s *t*-test or, when appropriate, Welch’s *t*-test. One-way ANOVA or, when appropriate, two-way ANOVA followed by Tukey’s multiple comparisons tests or Dunnett’s multiple comparisons tests was used to evaluate the difference among three or more groups. A *p*-value < 0.05 is considered statistically significant.

## Results

### Dynamic Changes in Myocardial Uchl1 Protein Expression and Ubiquitin Conjugates in Post-MI Mice

**FIGURE 1:**
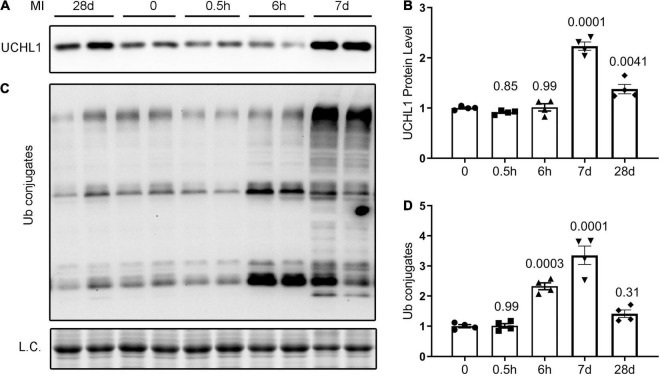
Dynamic changes in myocardial UCHL1 protein levels and ubiquitin (Ub) conjugates in post-MI mice. Female wild type mice were subjected to sham surgery or the ligation of the left anterior descending artery (LAD) to induce myocardial infarction (MI). Ventricular myocardium from the non-infarct area (peri-infarct plus remote area) were collected from the sham (0) and the MI mice at the indicated post-MI time points for total protein extraction. Representative images **(A)** and pooled densitometry data **(B)** of Western blot analyses for UCHL1. Representative images **(C)** and pooled densitometry data **(D)** of Western blot analyses for total Ub conjugates. L.C., loading control, which used the images from staining-free gel imaging technology; h, hour(s) post-MI; d, days post-MI; *n* = 4 mice per group; the *p*-value shown above each post-MI time point is derived from the pair-wise comparison between the post-MI time point and the sham control; one-way ANOVA followed by Tukey’s test.

Although a significant change in myocardial Uchl1 protein levels was not detected at 30-min or 6-h after LAD ligation (*p* = 0.85, 0.99), Western blot analyses showed that myocardial Uchl1 levels were significantly increased in the non-infarct area (boarder zone + remote area) both 7- and 28-days post-MI in CTL MI mice compared with sham controls (Figures 1A,C; *p* = 0.0001, 0.0041). A significantly greater level of myocardial Ub conjugates was detected at 6 h and 7 days post-MI (*p* = 0.0003, 0.0001), but the increases of myocardial Ub conjugates were subsided by 28 days post-MI (Figures 1B,D).

**FIGURE 2:**
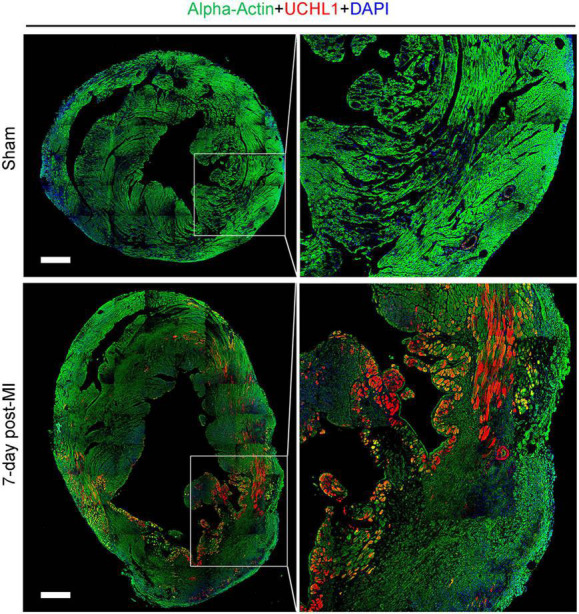
Post-MI myocardial upregulation of Uchl1 occurred primarily in the cardiomyocytes of the border zone in mice. Shown are representative micrographs of immunofluorescence staining for Uchl1 (red) and sarcomeric α-actin (green) in mouse hearts 7 days after LAD ligation or sham surgery. The sections were counter-stained with DAPI to visualize nuclear DNA (blue). The heart scale bar, 500 μm.

**FIGURE 3:**
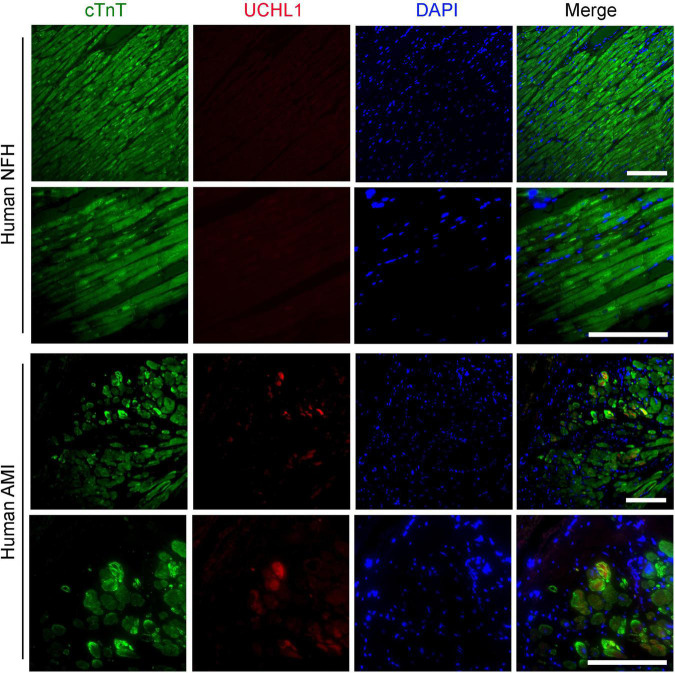
Post-MI myocardial upregulation of UCHL1 occurred primarily in the cardiomyocytes of the peri-infarct zone in humans. Paraffin-embedded and formalin-fixed ventricular myocardial sections (4-μm thick) from human non-failing donor hearts (NFH) or from the tissue cylinder core obtained from acute myocardial infarction (AMI) patients during the installation of a LV assisting device were subjected to deparaffinization, rehydration, antigen recovery, and indirect immunofluorescence staining for cardiac troponin T (cTnT) to identify cardiomyocytes (green) and for UCHL1 (red) as described in the Methods section. Nuclei are counter-stained with DAPI (blue). Shown are the representative confocal images. Scale bar, 100 μm.

Our double-immunofluorescence staining for sarcomeric α-actin and Uchl1 revealed that the upregulated Uchl1 in mouse myocardium is distributed primarily in the cardiomyocytes of the border zone in the MI hearts (Figure 2). To test whether this peri-infarct cardiomyocyte distribution of myocardial UCHL1 upregulation also occurs in humans, we performed double-immunofluorescence staining for UCHL1 and cTnT in myocardial samples from NFH and human hearts with AMI. We found that UCHL1 was increased exclusively in the cardiomyocytes (i.e., cTnT positive cells) of the peri-infarct area (Figure 3), confirming the findings from the animal study.

**FIGURE 4:**
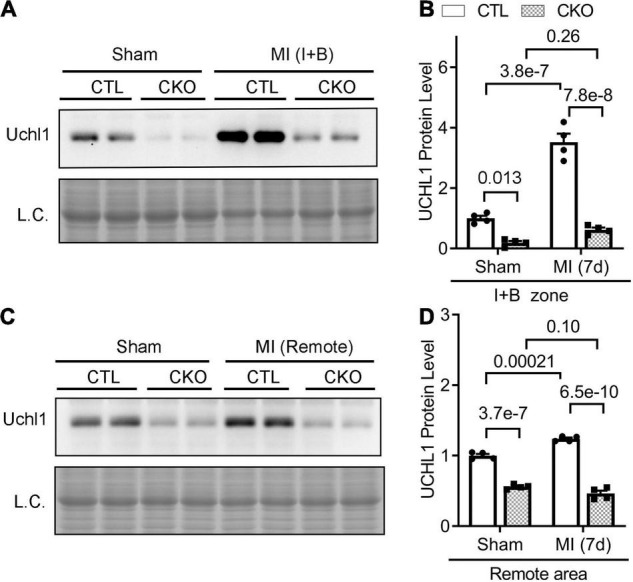
Upregulation of myocardial Uchl1 in post-MI mice occurs primarily in cardiomyocytes. Myh6-Cre transgenic (CTL) mice and mice with cardiomyocyte-restricted knockout of Uchl1 (CKO) at 10 weeks of age were subjected to sham surgery (Sham) or permanent LAD ligation (MI). For protein analyses, ventricular myocardium was sampled from the infarct and boarder (I + B) zone **(A,B)** or the remote area **(C,D)** of the post-MI mice or the equivalent areas of the sham control mice 7 days (7d) after LAD ligation. **(A,C)** Representative images of Western blot analyses for Uchl1 in the peri-infarct area (Peri-IF, **A**) and the remote zone **(C)**. L.C., loading control, which used the images from staining-free gel imaging technology. **(B,D)** Scatter plots superimposed by mean ± SEM to show the pooled densitometry data from four mice (two males and two females) per group. The *p*-value of the pair-wise comparison is shown above the bracket and is derived from two-way ANOVA followed by Tukey’s test.

We further examined myocardial Uchl1 protein levels of the infarct and boarder zone (I + B) and of the remote area 7 days post-MI using Western blotting. We found that the increases of Uchl1 proteins occurred at both zones, but the increases were more pronounced in the I + B zone (Figures 4A,B) than in the remote area (Figures 4C,D), compared with the corresponding areas of the sham control mice.

### Perinatal Uchl1-cko Did Not Induce Discernible Cardiac Abnormalities in Mice During at Least the First 14 Weeks of Age

**FIGURE 5:**
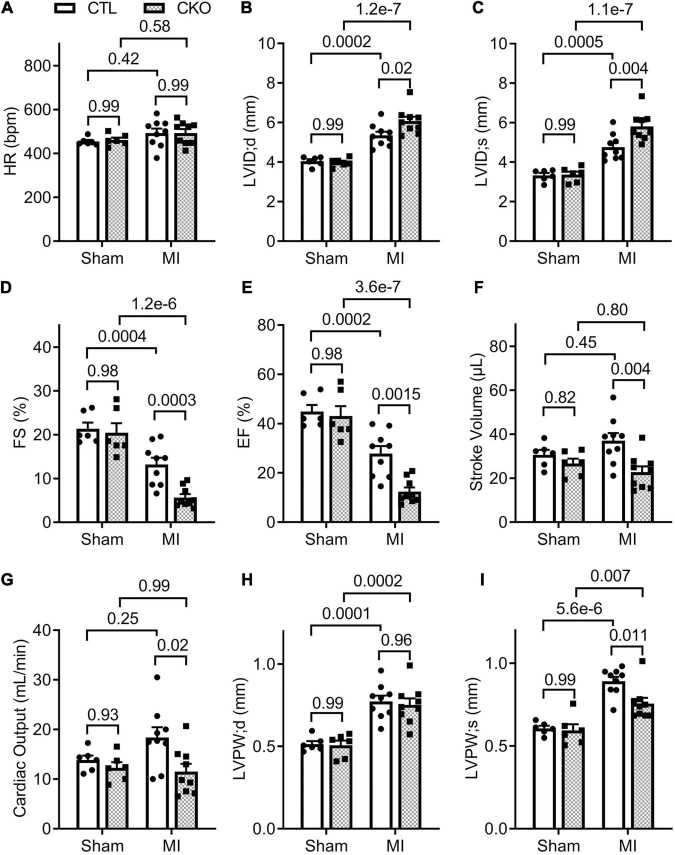
Echocardiographic assessments 28 days post-MI. CTL and CKO mice were subjected to sham surgery or LAD ligation as described in Figure 4. Parameters shown in panels **(A–I)** are derived from the B-mode guided M-model Echo performed 28 days post-MI. Each dot represents an individual mouse. For the sham groups, *n* = 6 mice (2 males + 4 females) for each genotype; for the MI groups, *n* = 9 mice (3 males + 6 females) for each genotype. The *p*-value of the pair-wise comparison shown above the bracket is derived from two-way ANOVA followed by Tukey’s tests.

**FIGURE 6:**
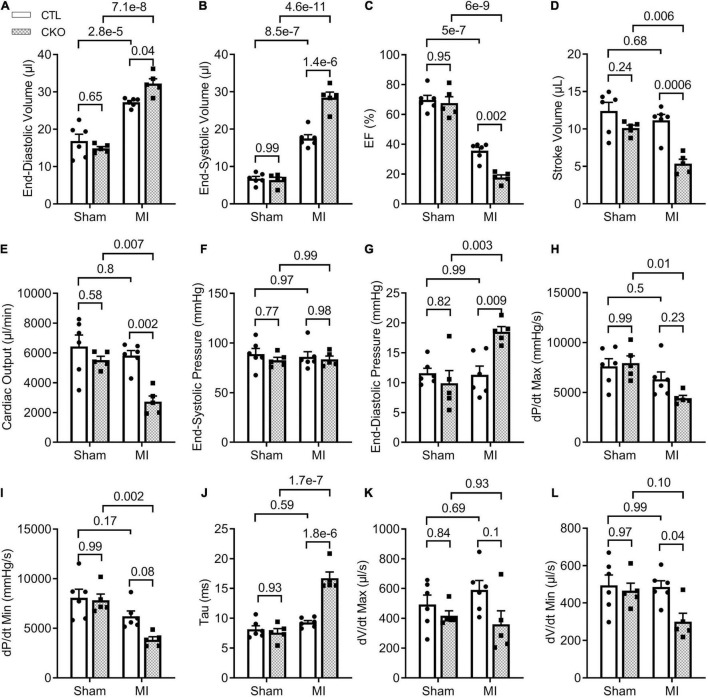
LV P-V loop analyses 28 days post-MI. CTL and CKO mice were subjected to sham surgery or LAD ligation as described in Figure 3. Parameters shown in panels **(A–L)** are derived from LV P-V loops collected 28 days post-MI. Each dot represents an individual mouse. For the CTL genotype, *n* = 6 mice (2 males + 4 females) for both Sham and MI groups; for the CKO genotype, *n* = 5 mice (2 males + 3 females) for both sham and MI groups. The *p*-value of the pair-wise comparison shown above the bracket is derived from two-way ANOVA followed by Tukey’s tests.

To assist in investigation into the physiological and pathophysiological significance of UCHL1 expressed in cardiomyocytes, we produced mice with perinatal Uchl1-cko, in which myocardial Uchl1 protein levels were reduced by approximately 85% compared to mice with the CTL genotype (Supplementary Figure 1A). Uchl1-cko did not alter myocardial protein levels of USP14 and Uchl5 (Supplementary Figure 2), two known DUBs that are reversibly associated with the 26S proteasome (30). Homozygous Uchl1-cko mice are viable and fertile; they were born in expected Mendelian ratios (Supplementary Figure 1B), indicating no embryonic lethality. No gross abnormalities were discerned for at least the first 14 weeks of age, the longest time closely observed so far. As shown in Figures 5, 6, neither Echo nor LV P-V relationship analyses detected cardiac morphometric and functional difference between CTL and Uchl1-cko mice in sham groups. These findings indicate that Uchl1 expressed in cardiomyocytes is not essential to perinatal cardiac development and, at least during the young adult phase, is not required for postnatal cardiac functioning under basal and mild stress conditions such as the sham surgery.

### Post-MI Myocardial Upregulation of Uchl1 Occurs Primarily in Cardiomyocytes

**FIGURE 7:**
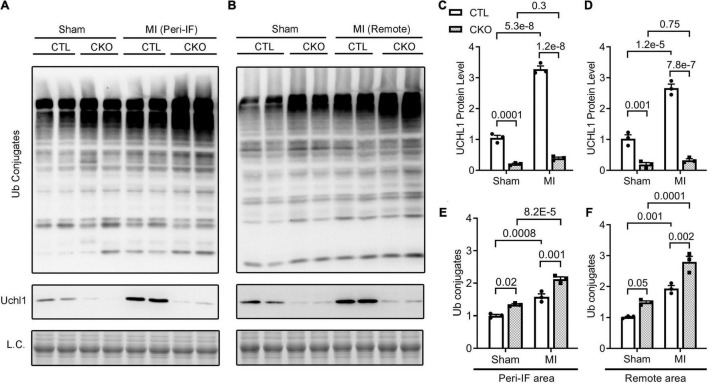
Western blot analyses for myocardial Uchl1 and total Ub conjugates in control (CTL) and Uchl1-cko (CKO) mice 28 days post-MI or sham surgery. The peri-infarct area (Peri-IF) and the remote area of the same hearts of the MI group as well as the equivalent areas of the sham group were analyzed. Shown are the representative images **(A,B)** and pooled densitometry data **(C–F)**. *N* = 3 mice (1 male + 2 females) for each group; the *p*-value of the pair-wise comparison shown above the bracket is derived from two-way ANOVA followed by Tukey’s tests.

We then subjected CTL and Uchl1-cko mice to permanent LAD ligation to induce MI and investigated the impact of Uchl1-cko on the myocardial Uchl1 upregulation 7 days post-MI. The MI-induced increases of Uchl1 in both the I + B zone and the remote area were completely abolished in Uchl1-cko mice (Figure 4). Similar findings were also obtained at 28-days post-MI (Figure 7). These results represent the first genetic evidence that post-MI myocardial upregulation of Uchl1 occurs primarily in cardiomyocytes.

### Post-MI Upregulation of Endogenous Ubiquitin Carboxyl-Terminal Hydrolase L1 Protects Against Maladaptive Cardiac Remodeling and Heart Failure

Since myocardial Uchl1 protein levels did not show significant changes during the initial hours post LAD ligation, we focused our study on the effect of Uchl1-cko on the long-term post-MI remodeling and the progression to heart failure using Echo and LV P-V relationship analyses. Echo at 7 days post-MI revealed that both the Uchl1-cko MI group and the CTL MI groups displayed comparable decreases in EF, FS, and LV anterior wall thickness at both end-diastole (LVAW;d) and end-systole (LVAW;s), as well as comparable increases in LV internal diameter at both end-diastole (LVID;d) and end-systole (LVID;s) (Supplementary Figure 3). By 28 days post-MI, despite significantly lower EF and FS, LV SV and CO were not significantly decreased in the CTL MI group compared with the CTL sham group (Figure 5), indicating that heart failure has not occurred yet in the CTL MI group at this time point. However, Echo revealed that the Uchl1-cko MI group displayed more pronounced decreases in EF and FS (*p* = 0.0015, 0.0003), smaller SV (*p* = 0.004) and CO (*p* = 0.02), greater LVID;d and LVID;s (*p* = 0.02, 0.004), and thinner LVPW;s (*p* = 0.011), compared with the CTL MI group although the LVPW;d was comparable between the two MI groups (Figure 5). These data indicate that post-MI LV chamber dilatation is exacerbated, and the functional compensation from the spared myocardium at the systole is compromised, by Uchl1-cko.

To further assess changes in LV function, especially diastolic function, we performed LV P-V relationship analyses 28 days post-MI (Supplementary Figure 4). In agreement with the Echo findings, a statistically significant difference was not discerned in any parameters of the LV P-V loop analyses between the CTL sham and the Uchl1-cko sham groups. The differences in EF, SV, and CO between the two MI groups as detected by Echo were confirmed by LV P-V relationship analyses (Figure 6). In agreement with the findings from the Echo-based comparison of LVID;d and LVID;s between the two MI groups, LV P-V loop analyses revealed that LV end-diastolic and end-systolic LV volumes were remarkably greater in the Uchl1-cko MI group than in the CTL MI group (*p* = 0.04, <0.00001). The LV end-systolic pressure did not differ significantly among the four groups and the LV end diastolic pressure (LVEDP) of the CTL MI group was comparable with the two sham groups; however, LVEDP of the Uchl1-cko group was remarkably elevated than that of the CTL MI group (*p* = 0.009). More importantly, the maximum velocities of LV pressure decrease (dP/dt-min) was lower (*p* = 0.08) and the time constant of exponential LV pressure decay (τ) was strikingly greater (*p* < 0.00001) in the Uchl1-cko MI group compared with CTL MI and Uchl1-cko sham groups although these parameters did not show significant differences between CTL MI and CTL sham groups yet at this time point (Figure 6). Differences in these key parameters reflecting the diastolic function status consistently indicate that Uchl1-cko impairs post-MI LV diastolic function.

**FIGURE 8:**
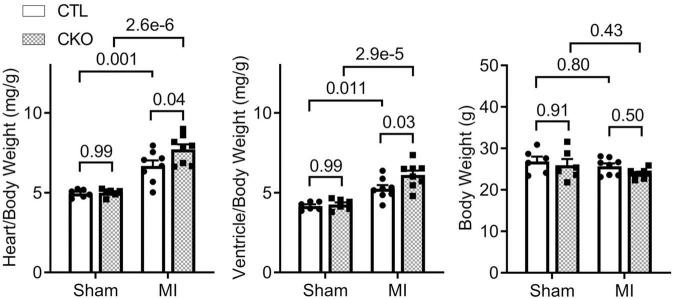
Gravimetric data collected at 28 days post-MI. For sham groups, *n* = 6 mice (2 males and 4 females) for each genotype; for MI groups, *n* = 8 mice (3 males + 5 females) for each genotype. The *p*-value of the pair-wise comparison shown above each bracket is derived from two-way ANOVA followed by Tukey’s tests.

Gravimetric analyses showed that the increases in the heart weight to body weight ratios and in the ventricular weight to body weight ratios were greater in Uchl1-cko MI mice than CTL MI mice (Figure 8; *p* = 0.04, 0.03), which is consistent with the more pronounced LV chamber dilatation but comparable LVPW;d in Uchl1-cko MI mice compared with CTL MI mice revealed by Echo at the same time point (Figure 5).

### Exacerbation of Myocardial Infarction-Induced Increases in Myocardial Ubiquitin Conjugates by Uchl1-cko

To explore the potential mechanism by which upregulation of UCHL1 suppresses post-MI maladaptive remodeling, we assessed changes in the steady-state myocardial ubiquitinated proteins in CTL and Uchl1-cko mice 28 days after sham or LAD ligation surgery using Western blot analyses (Figure 7). Myocardial levels of total Ub conjugates were higher in the Uchl1-cko sham group than in the CTL sham group (*p* = 0.02, 0.05). Compared with the respective sham group of the same genotype, myocardial Ub conjugates were significantly greater in the MI groups (*p* < 0.001, 0.0001 for peri-infarct area; *p* = 0.001, 0.0001 for remote area). And importantly, myocardial levels of Ub conjugates were remarkably higher in the Uchl1-cko MI group than in the CTL MI group (*p* = 0.001 for peri-infarct area, *p* = 0.002 for remote area). These experimental findings reveal that UCHL1 expressed in cardiomyocytes plays an indispensable role in myocardial proteostasis under a stress condition.

### Requirement of Ubiquitin Carboxyl-Terminal Hydrolase L1 for Myocardial and Cardiomyocyte Autophagic Flux

**FIGURE 9:**
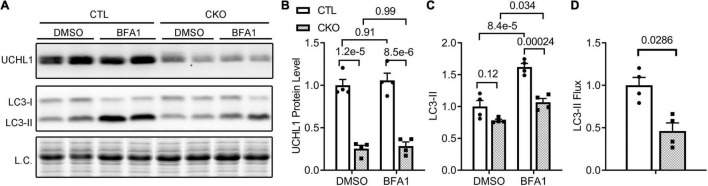
Cardiomyocyte-restricted Uchl1 deficiency decreases myocardial LC3-II flux in mice. Adult female Myh6-Cre transgenic (CTL) and Myh6-Cre:Uchl1*^flox/flox^* (CKO) mice were subjected to an intraperitoneal injection of bafilomycin-A1 (BFA1) or vehicle control (DMSO) and, 1 h later, ventricular myocardium was collected for total protein extraction and Western blot analysis for the indicated proteins. The total protein signals from the stain-free imaging were used as the in-lane loading control. Representative images **(A)** and pooled densitometry data **(B,C)** of Western blot analyses for the indicated proteins. Two-way ANOVA followed by Tukey’s test were used. **(D)** Relative myocardial LC3-II flux. The mean value of LC3-II levels in the DMSO treated group of a genotype was used as the baseline LC3-II value to subtract the LC3-II value of each BFA1 treated mouse in the same genotype groups, and the difference is regarded as the LC3-II flux for the mouse. The mean value of LC3-II flux values of the CTL group is then used to normalize all LC3-II flux values, the resultant relative LC3-II flux values are plotted in panel **(D)**. Two-tailed unpaired *t*-test was used for panel **(D)**.

Since myocardial Ub conjugates were increased in the Uchl1-cko sham group and an important pathway for the removal of ubiquitinated proteins is macroautophagy, we investigated the impact of Uchl1-cko on autophagic flux using the LC3-II flux assay. The tissue or cell levels of LC3-II proteins are a widely used indicator of autophagosome abundance (31); hence, the accumulation of LC3-II in cells/tissues by lysosome inhibition in a defined duration (i.e., LC3-II flux) reflects the autophagic flux in the cells/tissues (31). Compared with CTL mice, myocardial LC3-II flux in Uchl1-cko mice was significantly decreased (Figure 9).

**FIGURE 10:**
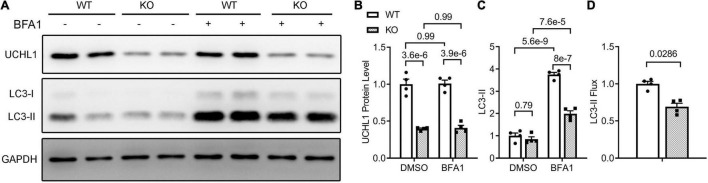
LC3-II flux assays in Uchl1-deficient and WT mouse ventricular cardiomyocytes. Cardiomyocytes isolated from 2-day old neonatal wild type (WT) or homozygous Uchl1-floxed (KO) mice were cultured for 48 h before infected with adenoviral vectors expressing the Cre recombinase (ad-Cre). Twenty-four hours later, these cells were treated with bafilomycin A1 (BFA1; 6 nM) or vehicle control (DMSO) for 24 h before they were harvested for total protein extraction. Representative images **(A)** and pooled densitometry data **(B,C)** of the Western blot analyses for the indicated proteins. **(D)** Relative LC3-II flux derived from the LC3-II flux assays. Related calculation and statistic methods are identical to what described in Figure 9.

To test whether the inhibition of myocardial autophagic flux by Uchl1-cko is cardiomyocyte-autonomous, we further examined the impact of Uchl1 deficiency on autophagic flux in cultured neonatal mouse cardiomyocytes, where Uchl1 knockout (KO) was achieved by coupling adenoviral delivery of *Cre* recombinase (Ad-Cre) with cultured neonatal cardiomyocytes from *Uchl1^flox/flox^* mice and the Ad-Cre treated WT cardiomyocytes served as the control. Results like that of the *in vivo* LC3-II flux assays were obtained from cultured neonatal mouse cardiomyocytes (Figure 10). The basal level of LC3-II was comparable between WT and Uchl1-KO cells, but lysosome inhibition by BFA1 induced significantly less increases of LC3-II in Uchl1-KO cells than in WT cells (Figure 10), indicating that LC3-II flux was significantly slower in Uchl1-KO cardiomyocytes than in WT cardiomyocytes.

## Discussion

Upregulation of myocardial UCHL1 was observed in human dilated cardiomyopathy and multiple animal models of cardiac disorders, but the pathophysiological significance of UCHL1 upregulation in the cardiomyocyte compartment has not been established. Although UCHL1 is highly expressed in the nervous tissue, here we provide the first genetic evidence that UCHL1 protein is also expressed in cardiomyocytes, this expression is upregulated in post-MI hearts, and this upregulation accounts for the entire increase of UCHL1 proteins in the post-MI myocardium. In other words, post-MI UCHL1 upregulation occurs exclusively in the cardiomyocyte compartment at both the border zone and remote area. Furthermore, we have uncovered that UCHL1 of cardiomyocytes is dispensable for perinatal cardiac development but Uchl1-cko impairs myocardial autophagic flux and that Uchl1-cko is well tolerated in young adult mice at baseline but exacerbates the post-MI disturbance of proteostasis and maladaptive cardiac remodeling. Therefore, this study demonstrates for the first time that UCHL1 supports autophagic flux in cardiomyocytes and that UCHL1 upregulation in cardiomyocytes in response to cardiac stress promotes cardiac proteostasis and protects against post-MI maladaptive cardiac remodeling and heart failure.

Ubiquitin carboxyl-terminal hydrolase L1 is expressed in an extremely high level in the nervous tissue, accounting for 1–2% of total proteins in the brain (4). This makes it very difficult to attribute changes of UCHL1 protein levels in myocardial homogenates to a specific cell type because there are nerves in myocardium and the nerve terminals are not evenly distributed, especially in a heart with MI. To this end, immunohistochemistry is certainly helpful in deciphering semi-quantitatively the relative changes among different cell types in the tissue and among different anatomic locations. Using Western blot analyses, Drobysheva et al. showed that UCHL1 proteins were increased similarly in both the LV and the right ventricle (RV) of mice at 1-week post-MI, the increases in both locations dwindled but remained significant at 4-week post-MI; however, no more increases were observed by 12-week post-MI (17). Interestingly, their immunofluorescence staining data revealed that the increase of UCHL1 was more appreciable in cardiomyocytes located primarily in the peri-infarct area (17), which seems to contradict the comparable magnitudes of increases between LV and RV because the MI was induced by LAD ligation and the infarct zone was located in the LV, away from the RV. Since immunofluorescence staining is semi-quantitative at best, the cardiomyocyte-enrichment of UCHL1 immunofluorescence does not necessarily rule out the possibility that UCHL1 might have been increased, albeit to a less extent, in other cell types of the myocardium as well. The conditional gene targeting strategy used in our study is known to knockout the target gene only in cardiomyocytes with a knockout efficiency of over 90% (32). Therefore, the reduction by 70–85% of myocardial Uchl1 protein levels in the Uchl1-cko mice compared with CTL mice at the baseline (Supplementary Figures 1, 2) indicates that UCHL1 is expressed in cardiomyocytes and UCHL1 expressed in the cardiomyocyte compartment constitutes the majority of UCHL1 proteins expressed in myocardium at baseline. Our findings further revealed that the increase in myocardial UCHL1 proteins did not occur either 30-min or 6-h after LAD ligation, but it was observed at both 7 and 28 days after LAD ligation. However, the increases in myocardial Ub conjugates in the MI hearts were detectable as early as 6 h after LAD ligation (Figure 1). This temporal relationship between the increases in Ub conjugates and in UCHL1 proteins suggests that the upregulation of UCHL1 might be a secondary response to an increased demand for deubiquitination in the ischemic myocardium and post-MI remodeling. Our further examination reveals that UCHL1 is upregulated in both the I + B zone and the remote area, but the increase in the former is much greater (Figures 2, 4). More importantly, the increases of Uchl1 proteins in both the I + B zone and the remote area were completely abolished in the Uchl1-cko mice (Figure 3), providing the first genetic and unequivocal evidence that post-MI upregulation of UCHL1 occurs primarily in the cardiomyocyte compartment. In agreement with these findings, our immunofluorescence staining revealed that the post-MI increases of myocardial UCHL1 proteins were concentrated in the cardiomyocytes of the peri-infarct area in the mouse model (Figure 2), which is supported by a prior report (17). Notably, we have confirmed this finding for the first time in human post-MI myocardial specimen (Figure 3).

Another major contribution of the present study is the discovery that UCHL1 is required for autophagic flux in cardiomyocytes, as supported by that LC3-II flux in Uchl1-cko mouse myocardium and in cultured neonatal Uchl1-KO mouse cardiomyocytes were significantly reduced (Figures 9, 10). The decrease in autophagic flux is probably caused by impaired formation, rather than impaired removal, of autophagosomes. This is because the basal LC3-II levels were not significantly increased by the ablation of *Uchl1* in cardiomyocytes in mice or in cultures, whereas ablation of *Uchl1* in cardiomyocytes significantly attenuated the accumulation of LC3-II by inhibition of lysosomes with BFA1 both in mice (Figure 9) and in cultured mouse cardiomyocytes (Figure 10). The reduction of autophagic flux due to cardiomyocyte-restricted UCHL1 deficiency has a modest but discernible effect on myocardial proteostasis in the shame surgery control group as reflected by the moderate but statistically significant increase of myocardial Ub conjugates in the Uchl1-cko sham group compared with the CTL sham group 28 days after the sham surgery (Figure 7). However, this seems to be well tolerated by at least young adult mice because no significant differences in LV morphometry and cardiac function were discerned by either Echo (Figure 5) or LV P-V relationship analyses (Figure 6) between the two sham groups.

The detrimental effects of this UCHL1-cko induced proteostasis impairment become apparent during post-MI remodeling. The impairment of proteostasis by MI as indicated by the increases of myocardial Ub conjugates in the MI hearts was further exacerbated by Uchl1-cko (Figure 7). Echo performed 7 days after LAD ligation revealed that MI had caused marked LV chamber dilatation, reduction of LV anterior wall thickness, significant decreases in EF and FS, but these changes were comparable between the CTL MI and the Uchl1-cko MI groups (Supplementary Figure 3). These suggest that Uchl1-cko probably does not alter the infract size and the detrimental effect of UCHL1 deficiency at this early post-MI stage, if any, can be well compensated. By 28 days post-MI, however, drastic differences in both LV morphometry and functioning were detected between the two MI groups. LV chamber dilatation as reflected by the increases in LV end-diastolic diameter (LVID;d; as measured by Echo) and LV end-diastolic volume (as measured by P-V loop), and the reduction of EF and FS were more pronounced in the Uchl1-cko MI group than in the CTL MI group at 28 days post-MI. As a result, significant decreases in SV and CO were observed in the Uchl1-cko MI group but not in the CTL MI group yet at this time point (Figures 5, 6). Cardiomyocyte-restricted UCHL1 deficiency exacerbates the impairment of both systolic and diastolic function during the post-MI remodeling, which is well supported by our data from both Echo and LV P-V relationship analyses (Figures 5, 6). These findings are in stark contrast to a recent report which showed a protective effect of systemic UCHL1 inhibition by LDN-57444 against post-MI myocardial fibrosis and LV malfunction in mice (15), suggesting that inhibition of UCHL1 in the non-cardiomyocyte compartment or an off-target effect of LDN-57444 might play an important role in protecting the post-MI heart by this pharmacological compound.

Consistent with the comparable increase of LVPW;d and a greater increase of LVID;d in Uchl1-cko MI group compared with the CTL MI group (Figure 5), significantly greater increases in the heart weight to body weight ratios and in the ventricular weigh to body weight ratios were observed in the Uchl1-cko MI group compared with the CTL MI group (Figure 8), indicating that cardiomyocyte-restricted UCHL1 deficiency exacerbates post-MI eccentric cardiac hypertrophy. To some extent, these findings are in contrast to the previously reported studies showing that systemic inhibition of UCHL1 using both genetic or pharmacological approaches significantly suppressed pressure-overloaded cardiac hypertrophy (20, 22), suggesting that the blood pressure reduction property of chronic systemic UCHL1 inhibition might have played a greater role in the observed suppression of cardiac hypertrophy than originally thought (22).

To our best knowledge, this represents the first study on cardiomyocyte-restricted loss of function of UCHL1. It demonstrates that although loss of function of UCHL1 in cardiomyocytes is well tolerated at baseline, it is detrimental to cardiac proteostasis, maladaptive cardiac remodeling and LV malfunction after MI. Cardiomyocyte-restricted inhibition of UCHL1 yields starkly different effects on post-MI hearts from the previously reported systemic UCHL1 inhibition; hence, it will be very interesting and important to investigate the effects of the inhibition of UCHL1 selectively in the non-cardiomyocyte compartments on post-MI remodeling and other cardiac disorders. Consistent with our findings, it is expected that UCHL1 gain of function should show beneficial effects on post-MI remodeling. Indeed, in a companion study that is under the consideration elsewhere, we were able to demonstrate that overexpression of murine UCHL1 initiated at the neonatal stage *via* adeno associated virus mediated cardiomyocyte-restricted gene delivery (AAV9-cTNT-m-UCHL1) confers significant resistance to MI injury (33).

Ubiquitin carboxyl-terminal hydrolase L1 inhibition is being experimentally tested for its antifibrotic property (12–15) and explored as a potentially new anti-malignancy strategy (9). The findings of the present study implicate that the UCHL1 inhibition probably can be tolerated by normal hearts because the complete loss of function of UCHL1 in cardiomyocytes did not show discernible abnormality; however, such a treatment should be watched for cardiotoxicity if the subject has cardiac co-morbidities because UCHL1 deficiency does impair cardiac proteostasis when the heart is under a stress condition such as ischemic heart disease.

## Data Availability Statement

The original contributions presented in the study are included in the article/Supplementary Material, further inquiries can be directed to the corresponding author.

## Ethics Statement

The studies involving human participants were reviewed and approved by the Institutional Review Board of the University of Minnesota. The patients/participants provided their written informed consent to participate in this study. The animal study was reviewed and approved by the University of South Dakota Institutional Animal Care and Use Committee and the University Laboratory Animal Resources of The Ohio State University.

## Author Contributions

XW, PW, and JL conceived the study, designed the experiments, analyzed the data, and wrote the manuscript. PW, MC, BG, HZ, BY, YL, and FL collected the data and participated in data analyses. YL provided critical reagent before its publication. All authors have read the manuscript and approved for its submission for publication.

## Conflict of Interest

The authors declare that the research was conducted in the absence of any commercial or financial relationships that could be construed as a potential conflict of interest.

## Publisher’s Note

All claims expressed in this article are solely those of the authors and do not necessarily represent those of their affiliated organizations, or those of the publisher, the editors and the reviewers. Any product that may be evaluated in this article, or claim that may be made by its manufacturer, is not guaranteed or endorsed by the publisher.
