# Supplementary material for: Ubiquitin Carboxyl-Terminal Hydrolase L1 of Cardiomyocytes Promotes Macroautophagy and Proteostasis and Protects Against Post-myocardial Infarction Cardiac Remodeling and Heart Failure
Source: Front Cardiovasc Med. 2022 Apr 7;9:866901. doi: 10.3389/fcvm.2022.866901 (PMC9021418; doi:10.3389/fcvm.2022.866901)
Supplement: Supplementary file 1 [file Data_Sheet_1.PDF]

Supplementary Materials to

UCHL1 of cardiomyocytes promotes macroautophagy and proteostasis and protects against post-myocardial infarction cardiac remodeling and heart failure

Penglong Wu[1,2], Yifan Li[1], Mingqi Cai[1], Bo Ye[3], Bingchuan Geng[4], Faqian Li[5], Hua Zhu[4], Jinbao Liu[6], Xuejun Wang[1*]

From [1]Division of Basic Biomedical Sciences, University of South Dakota Sanford School of Medicine, Vermillion, SD 57069, USA; [2]Department of Cardiology, Xiamen Cardiovascular Hospital of Xiamen University, Xiamen University School of Medicine, Xiamen, Fujian, China; [3]Department of Cardiology, University of Minnesota School of Medicine, Minneapolis, MN55455, USA; [4]Department of Surgery, The Ohio State University Wexner Medical Center, Columbus, OH 43210, USA; [5]Department of Laboratory Medicine and Pathology, University of Minnesota School of Medicine, Minneapolis, MN55455, USA; [6]Guangzhou Municipal and Guangdong Provincial Key Laboratory of Protein Modification and Degradation, School of Basic Medical Sciences, Guangzhou Medical University, Guangzhou 511436, China.

Supplementary Table 1. Source of Antibodies

Target antigen	Vendor or Source	Catalog #	Working Concentration	Persistent ID/ URL
Actin, α-sarcomeric	Sigma Aldrich	A2172	IF, 1:600	https://www.sigmaaldrich.com/US/en/product/sigma/a2172
Uchl1	SantaCruz	sc-271639	WB, 1:1000	https://www.scbt.com/p/uch-l1-antibody-c-4
UCHL1	Cell Signaling Technology	#13179	IF, 1:200	https://www.cellsignal.com/products/primary-antibodies/uchl1-d3t2e-xp-rabbit-mab/13179
cTnT	Thermo Fisher	MA5-12960	IF, 1:200	https://www.thermofisher.com/antibody/product/Cardiac-Troponin-T-Antibody-clone-13-11-Monoclonal/MA5-12960
USP14	Bethyl Laboratories, Inc.	A300-920A	WB, 1:1000	https://www.thermofisher.com/antibody/product/USP14-Antibody-Polyclonal/A300-920A
Ubiquitin	Sigma	SAB4503053-100UG	WB, 1:10000	https://www.sigmaaldrich.com/US/en/product/sigma/sab4503053
LC3	Cell Signaling Technology	#2775S	WB, 1:1000	https://www.cellsignal.com/products/primary-antibodies/lc3b-antibody/2775
UCHL5	Abcam	ab133508	WB, 1:1000	https://www.abcam.com/uch37-antibody-ep4897-ab133508.html
GAPDH	Sigma-Aldrich	G8795	WB, 1:1000	https://www.sigmaaldrich.com/catalog/product/sigma/g8795?lang=en&region=US

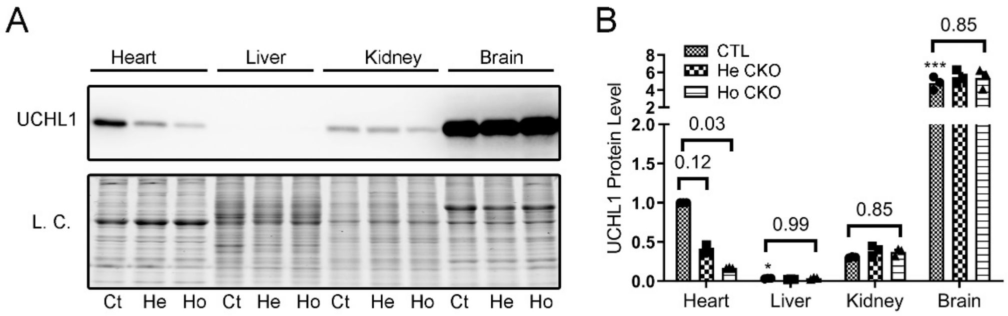

C

Myh6-Cre::Uchl1$^{FL/FL}$ x Uchl1$^{FL/+}$ or Myh6-Cre::Uchl1$^{FL/+}$ x Uchl1$^{FL/FL}$

⇩

Off-spring genotypes	Uchl1$^{FL/+}$	Uchl1$^{FL/FL}$	Myh6-Cre::Uchl1$^{FL/+}$	Myh6-Cre::Uchl1$^{FL/FL}$	
Number of pups	25	23	22	25	95
%	26.32%	24.21%	23.15%	26.32%	100.00%

Supplementary Figure 1. A and **B**, Western blot analysis for Uchl1 in main organs of mice with the indicated genotypes: control (CTL; Myh6-Cre, Uchl1$^{FL/+}$, and Uchl1$^{FL/FL}$), Myh6-Cre::Uchl1$^{FL/+}$ (He CKO), and Myh6-Cre::Uchl1$^{FL/FL}$ (Ho CKO). L.C., loading control, segment of the stain-free total protein image. One-way ANOVA followed by Tukey's tests. **C**, Genotype distribution of the off-springs from the indicated breeding strategies.

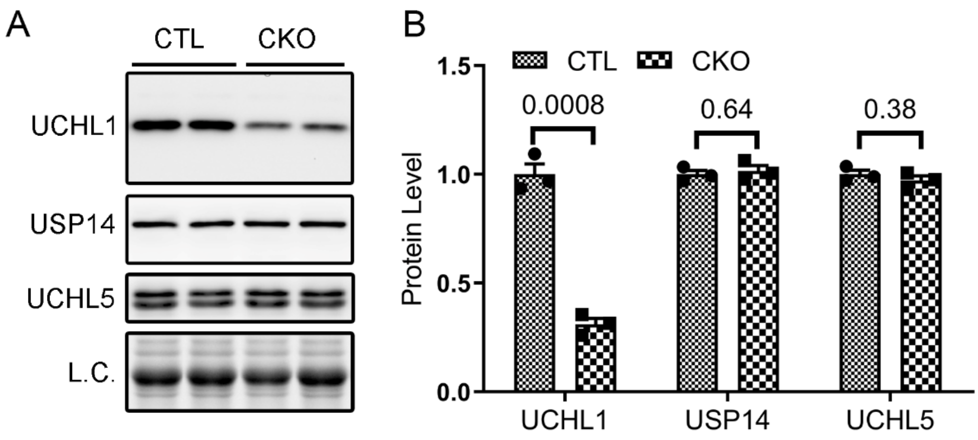

Supplementary Figure 2. Perinatal Uchl1-cko did not affect myocardial expression of USP14 and Uchl5 in mice. Shown are the representative images (**A**) and pooled densitometry data (**B**) of Western blot analyses for the indicated proteins in the ventricular myocardium of you adult mice with the indicated genotypes. CTL, Myh6-Cre transgenic; CKO, Myh6-Cre^{TG}::Uchl1$^{FL/FL}$. Each dot represents a mouse; Welch's t-test.

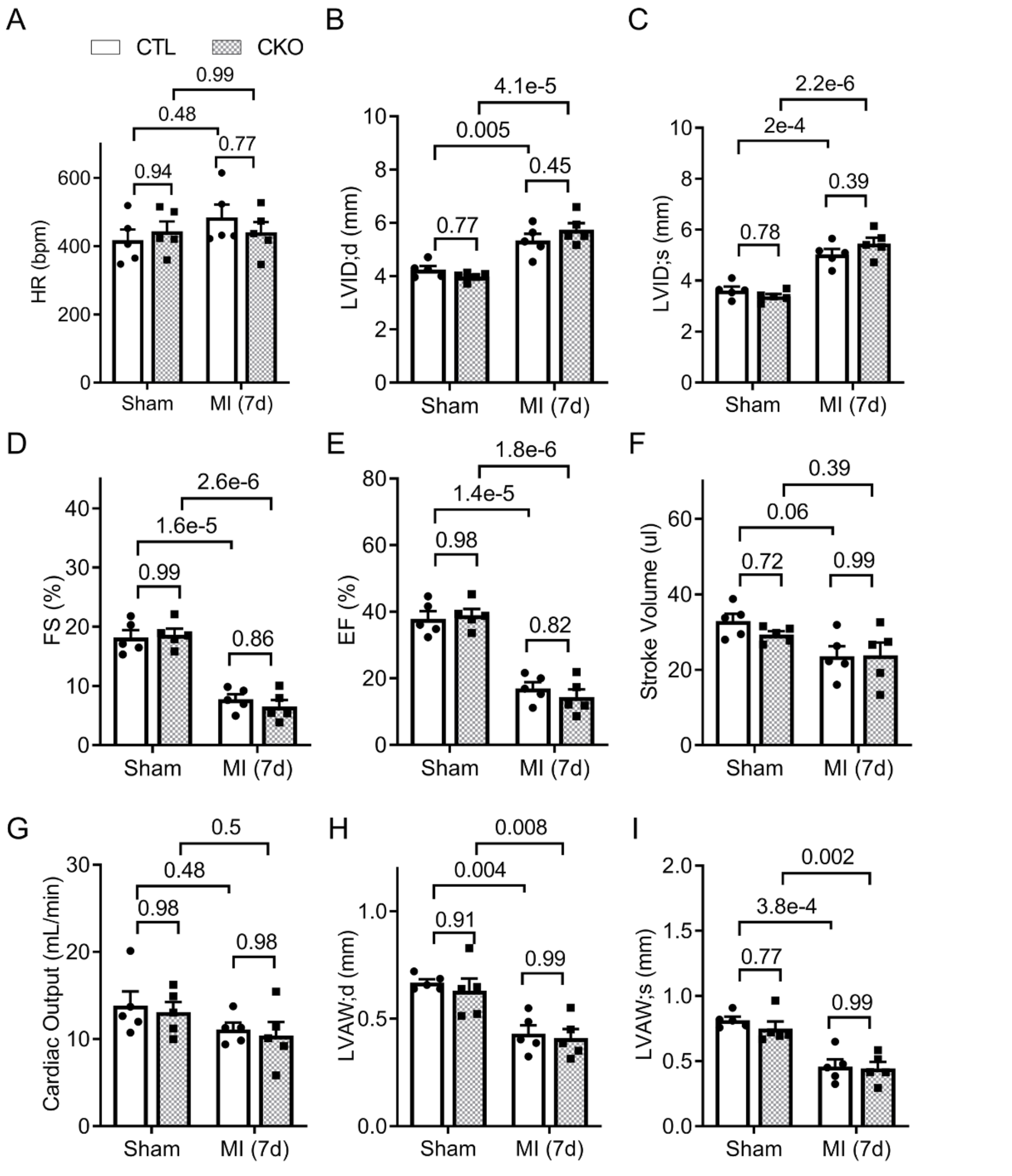

Supplementary Figure 3. Echocardiography data from CTL and homozygous Uchl1-cko mice 7 days after LAD ligation (MI) or sham surgery (Sham). N=5 mice (2 males + 3 females) per group.

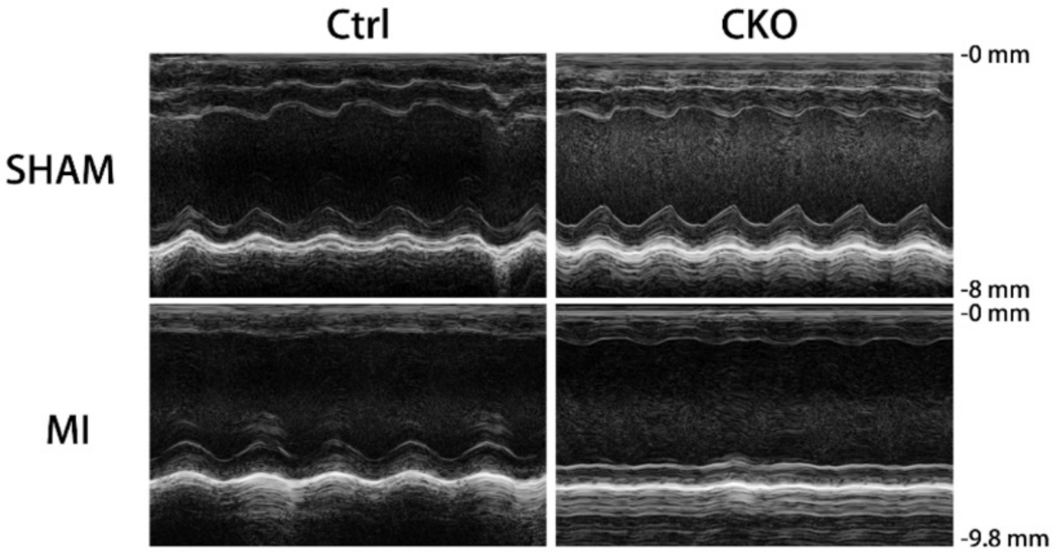

Supplementary Figure 4. Representative echocardiograms collected from control (Ctrl) and homozygous Uchl1-cko (CKO) mice at 28 days after LAD ligation (MI) or sham surgery (SHAM).

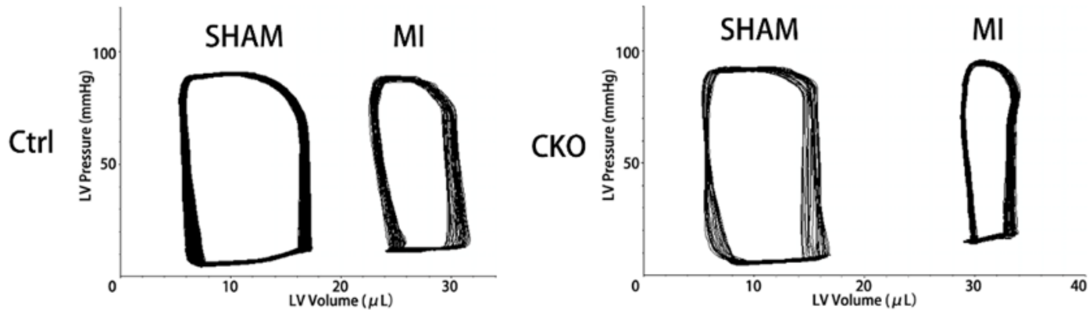

Supplementary Figure 5. Representative LV P-V loop images collected from control (Ctrl) and homozygous Uchl1-cko (CKO) mice at 28 days after LAD ligation (MI) or sham surgery (SHAM).
